# Pharmacokinetic Behavior and Pharmacokinetic/Pharmacodynamic Integration of Danofloxacin Following Single or Co-Administration with Meloxicam in Healthy Lambs and Lambs with Respiratory Infections

**DOI:** 10.3390/antibiotics10101190

**Published:** 2021-09-30

**Authors:** Mehmet Nihat Ural, Kamil Uney

**Affiliations:** 1 Department of Pharmacology and Toxicology, Faculty of Veterinary Medicine, University of Selcuk, 42031 Konya, Turkey; nihatural@yahoo.com; 2 Pendik Veterinary Control Institute, Bati Mah. Yunus Cad. 2/1, Pendik, 34890 Istanbul, Turkey

**Keywords:** danofloxacin, pharmacokinetics, pharmacodynamics, meloxicam, respiratory infection

## Abstract

The aim of this study was to determine the pharmacokinetics and pharmacodynamics of danofloxacin (DAN; 6 mg/kg) following subcutaneous administration alone or co-administration with meloxicam (MLX; 1 mg/kg) in healthy lambs and lambs with respiratory infections. The study was carried out using a total of four groups: HD (healthy; *n* = 6) and ID (infected; *n* = 7) groups who were administered DAN only, and HDM (healthy; *n* = 6) and IDM (infected; *n* = 7) groups who were administered DAN and MLX simultaneously. The plasma concentrations of DAN were determined using high-performance liquid chromatography–UV and analyzed by the non-compartmental method. DAN exhibited a similar elimination half-life in all groups, including both the healthy and infected lambs. The total clearance in the HDM, ID and IDM groups and volume of distribution in the HDM and IDM groups were significantly reduced. MLX in the IDM group significantly increased the area under the curve (AUC) and peak concentration (C_max_) of DAN compared to the HD group. The *Mannheimia haemolytica*, *Escherichia coli*, and *Streptococcus* spp. strains were isolated from bronchoalveolar lavage fluid samples of the infected lambs. When co-administration with meloxicam, DAN at a 6 mg/kg dose can provide optimum values of *ƒ*AUC_0–24_/MIC (>56 h) and *ƒ*C_max_/MIC (>8) for susceptible *M. haemolytica* isolates with an MIC_90_ value of 0.25 µg/mL and susceptible *E. coli* isolates with an MIC value of ≤0.125 µg/mL.

## 1. Introduction

Respiratory diseases, which are common in all countries where lamb breeding is carried out, are considered to be complex diseases that include interactions between multiple agents such as the host’s immune system and physiological state, viruses, bacteria, *Mycoplasma*, and environmental factors. They cause serious financial losses due to low body weight gain, treatment costs, and death [1,2,3]. Bacterial agents frequently isolated from lambs with pneumonia are *Mannheimia haemolytica* and *Pasteurella multocida* [4,5]. Antibiotics are used to treat acute pneumonia, to reduce the effects of chronic nonprogressive pneumonia, and to treat the remainder of the flock to prevent more deaths from acute pneumonia. The quick detection of infected lambs is very important for the success of antibiotic treatment [3,6]. The antibiotic selection for the treatment of infection depends on many factors, including the infective microorganism, the susceptibility of the microorganism to antibiotics, the site of infection, local factors (pH, inoculum size, etc.), host factors, the potential side effects of antibiotics, and antibiotic resistance. Following the selection of the ideal antibiotic, the pharmacokinetic and pharmacodynamic data of the drug should be determined to establish the most appropriate dosage regimen [7,8].

Danofloxacin, a third-generation synthetic fluoroquinolone developed for use in veterinary medicine, is approved for the treatment of respiratory infections in cattle, chickens, and pigs [9,10]. Danofloxacin reaches a 4–5 times higher concentration in the lung tissue than in the plasma and has activity against most Gram-negative bacteria, such as *P. multocida*, *M. haemolytica*, *Histophilus somni*, and Enterobacterales (*Escherichia coli, Klebsiella,* and *Salmonella*), and some Gram-positive bacteria, such as *Staphylococcus aureus* and *Streptococcus pneumoniae* [11,12,13,14]. Danofloxacin has a very wide distribution volume in sheep and goats due to having high lipid solubility, low binding to plasma proteins, and being a substrate for ATP-dependent efflux transporters [15,16]. The clinical efficacy of danofloxacin has been established in sheep and cattle with respiratory infections [17,18].

Meloxicam, a non-steroidal anti-inflammatory drug (NSAID) of the oxicam class, is used to alleviate the symptoms of acute respiratory infection with appropriate antibiotic therapy in cattle [19]. In Canada, Australia, and New Zealand, it is approved for use in the treatment of pain and inflammation in sheep [20]. It has been determined that meloxicam has a stronger anti-inflammatory effect by reducing the synthesis of tumor necrosis factor (TNF)-alpha, a cytokine with proinflammatory effects, as well as inhibiting the cyclooxygenase (COX)-2 enzyme in respiratory infections [21,22].

In respiratory infections, the host’s excessive inflammatory response plays an important role in the pathogenesis of the disease [23]. Therefore, the use of NSAIDs is recommended against the harmful effects of inflammation in the treatment of pneumonia [24,25]. It has been reported that when NSAIDs are used in combination with antibiotics compared to antibacterial therapy alone, clinical findings improve faster in respiratory infection [25,26,27]. However, pathophysiological changes in respiratory infections and antibiotics–NSAID interactions resulting from concurrent drug administration during treatment may cause unpredictable changes in the pharmacokinetics of antibiotics. These changes may lead to the development of resistance to antibiotics, the emergence of undesirable effects, and a suboptimal level of drug exposure, leading to failure in the treatment of infection [28,29]. Although there are many studies investigating the effects of NSAIDs on the pharmacokinetics of antibiotics in healthy animals [30,31,32,33,34,35,36], studies conducted in the case of disease are limited [37,38,39]. We hypothesized that the effect of meloxicam on the pharmacokinetics of danofloxacin will change with the systemic inflammatory response caused by respiratory infection in lambs, and that the results obtained will contribute to the rational use of danofloxacin.

Therefore, the current study was conducted to provide the following information: (1) the subcutaneous (SC) pharmacokinetics of danofloxacin (6 mg/kg) administered alone or simultaneously with meloxicam (1 mg/kg; SC) in healthy lambs and lambs with respiratory infections, (2) isolation of bacterial agents from bronchoalveolar lavage (BAL) fluid samples of lambs with respiratory infections, (3) determination of the minimum inhibitory concentration (MIC) of danofloxacin for isolated bacterial agents, and (4) estimation of the surrogate markers (*ƒ*C_max_/MIC and *ƒ*AUC/MIC) of antibacterial activity by integration of the pharmacokinetic parameters obtained in vivo with in vitro MIC data.

## 2. Materials and Methods

### 2.1. Animals

This study was carried out on lambs with healthy and respiratory infections, who had not received any medication in the month prior to administration. During the study, lambs were fed with commercial feed (CP-5621, Ankara) twice a day, and alfalfa hay, grass hay, and water were given ad libitum. All procedures on animals were approved by the local ethics committee (Faculty of Veterinary Medicine, University of Selcuk, Konya, Turkey).

### 2.2. Clinical Examination and Creating Groups

Before the study, lamb farms were screened for the presence of respiratory infections. Lambs (Kivircik breed, 3–4 months old, *n* = 174) on a farm (TR4223131, Kadınhanı/Konya) in whom respiratory infections were detected were examined for appetite, coughing, nasal and eye discharge, increased respiratory rate, rectal temperature, and lung sounds. The clinical scoring parameters (scores of 0–3) reported by Christodoulopoulos et al. were used to determine the disease and its severity [40]. Lambs (*n* = 76) determined to be healthy upon clinical examination were taken into clean pens and were observed clinically for 7 days. To determine the pharmacokinetics of danofloxacin, 12 lambs were randomly selected from the healthy lambs following clinical examination and placed into two pens of 20 m^2^ with 6 lambs each, who were then numbered using oil paint.

The lambs (*n* = 98) with respiratory infections were placed into different pens according to their clinical scores (scores of 1–3) and numbered using oil paint. Bronchoalveolar lavage (BAL) fluid samples from these lambs were taken for the isolation and identification of bacterial agents caused respiratory infections. In infected lambs, an increased respiratory rate (46% with >48 breaths/min); abnormal findings, including abnormal sounds (audible, predominantly antero-ventrally, and throughout the entire lung field) under lung auscultation (92%); coughing (48%); nasal discharge (42%), including scanty serous, abundant serous, and mucopurulent discharge; eye discharge (27%), including scanty serous, abundant serous, and purulent discharge; and increased rectal temperature (46% with >40 °C) were revealed with a detailed clinical examination. To determine the pharmacokinetics of danofloxacin, 14 lambs were randomly selected from the infected lambs (*n* = 23) with a score of 3, coughing, an increased respiratory rate (>55 breaths/min), and an increased rectal temperature (>40.5 °C) and were placed into two pens of 20 m^2^ with 7 lambs each.

### 2.3. Pharmacokinetic Study

The age, body weight, rectal temperature, and respiration rate of the healthy lambs and those with respiratory infections selected to determine the pharmacokinetics of danofloxacin are presented in Table 1. The pharmacokinetic study was carried out using a total of 4 groups: HD (healthy; *n* = 6) and ID (infected; *n* = 7) groups, who were administered danofloxacin only, and HDM (healthy; *n* = 6) and IDM (infected; *n* = 7) groups, who were administered danofloxacin and meloxicam simultaneously. Danofloxacin (6 mg/kg, Advocin 180, 180 mg/mL, injection solution, Zoetis, France) and meloxicam (1 mg/kg, Maxicam X4, 20 mg/mL, injection solution, Sanovel, Turkey) were administered subcutaneously to the right and left axillary regions of the lambs, respectively. Blood samples (2 mL) were collected in lithium heparin-containing anticoagulant tubes via a catheter from the right jugular vein before (0 min) drug administration and at 0.25, 0.3, 0.45, 1, 2, 4, 6, 8, 10, 12, 18, 24, and 48 h after drug administration. All blood samples were centrifuged at 3000× *g* for 10 min, and the harvested plasma were stored at −70 °C until analysis.

#### 2.3.1. Danofloxacin Analysis

The plasma concentration of danofloxacin was analyzed using the high-performance liquid chromatography (HPLC)–UV system (Shimadzu, Tokyo, Japan) as per a previously reported method [41,42,43,44]. First, 200 μL of acetonitrile was added to 200 μL of the plasma sample. The mixture was vortexed for 30 s and centrifuged at 9167× *g* for 10 min at 15 °C. Then, 20 μL of supernatant was injected into the HPLC–UV system, comprising a pump (LC-20AT controlled CBM-20A), degasser (DGU-14A), autosampler (SIL-20A), column oven (CTO-10A), and ultraviolet detector (SPD-10A), which was set to 278 nm. Chromatographic separation was performed using a Gemini ™ C18 column (250 × 4.6 mm; internal diameter, 5 µm; Phenomenex, Torrance, CA, USA). The column oven and autosampler temperatures were kept at 30 and 22 °C, respectively. The mobile phase comprised orthophosphoric acid of 0.4% in water, including 0.4% triethylamine and acetonitrile (87:13, *v/v*), pumped at a flow rate of 1 mL/min. HPLC system control and data analysis were performed with LC solution software (Shimadzu, Japan). To prepare the calibration standards (0.04–10 μg/mL) and the quality control samples (0.05, 0.5, and 5 μg/mL), control plasma from animals that had received no treatment was spiked with working standards of danofloxacin (98%, analytical purity; Sigma-Aldrich, St. Louis, MO, USA) prepared in water. The calibration standards and quality control samples were run with each assay. The linearity of the standard curve created with the calibration standards was *r*^2^ > 0.999. The lower limit of quantitation (LLOQ) was 0.04 μg/mL with a coefficient of variation of less than 20% and a bias of ±15%. To determine the recovery, precision, and accuracy of the assay, five replicates for quality control samples were tested for five consecutive days. The recovery from the plasma was >91%. The percent coefficient of variation for the intra- and interday precision was <7.9%, while the percent bias for the intra- and interday accuracy of the assay was ±5.3%.

#### 2.3.2. Pharmacokinetic Analysis

WinNonlin 6.1.0.173 software (Pharsight Corporation, Scientific Consulting Inc., Sunnyvale, CA, USA) was used to plot the plasma concentration–time curve of danofloxacin for each lamb and to calculate the pharmacokinetic parameters by the non-compartmental method using the formulae described by Gibaldi and Perrier [45]. After SC administration, the elimination rate constant (ʎz), terminal elimination half-life (t_1/2ʎz_), area under the curve (AUC), total body clearance (Cl_T_/F), volume of distribution (Vd_area_/F), and mean residence time (MRT) were calculated. The volume of distribution at a steady state (Vd_ss_/F) after SC administration was calculated as MRT * Cl_T_/F. The AUC was determined by the linear up/log down method. The ʎz was determined from the linear portion of the terminal phase by linear regression analysis. The peak concentration (C_max_) and the time to reach the C_max_ (T_max_) were determined by direct observation from the individual plasma concentration–time curves plotted for each lamb that received danofloxacin.

#### 2.3.3. In Vitro Protein Binding Assay of Danofloxacin

In plasma samples obtained from healthy lambs and lambs with respiratory infections, the plasma protein binding ratio of danofloxacin alone or in combination with meloxicam was evaluated using ultrafiltration [46]. Studies were carried out at physiologic temperature and pH (7.4). Pooled plasma was separately collected from healthy lambs (*n* = 12, HD and HDM groups) and lambs (*n* = 14, ID and IDM groups) with respiratory infections that had received no drug treatment. Then, the obtained plasma samples were divided into 4 groups: PBHD and PBID groups who were spiked with danofloxacin only, and PBHDM and PBIDM groups who were spiked with danofloxacin and meloxicam simultaneously. Plasma samples were spiked with danofloxacin and meloxicam to make a 0.05, 0.5, and 5 μg/mL concentration. Three replicates of each concentration of danofloxacin were made. To achieve equilibrium between drugs and plasma proteins, the spiked plasma samples were incubated at 37 °C for 30 min prior to ultrafiltration. Samples of 1 mL were transferred to an Amicon Ultra Centrifugal Filter (Ultracel 10 kD; Millipore Corporation, Bedford, MA, USA) and centrifuged at 4000× *g* for 15 min. The ultrafiltrate was analyzed directly by HPLC for determination of free danofloxacin concentrations. The protein binding of danofloxacin was determined the following formula: Protein binding (%) = [(Total drug−Free drug)/Total drug] × 100. In addition, a preliminary study was performed to identify danofloxacin nonspecific binding to the ultrafiltration device.

#### 2.3.4. Statistical Analysis

All values for the pharmacokinetic parameters and pharmacokinetic/pharmacodynamic (PK/PD) data are expressed as the geometric mean (min–max). Other values are presented as the mean ± SD. The normality of the data distribution was assessed with the Shapiro–Wilk test and the homogeneity of variance with the Levene’s test. A two-way between groups ANOVA was used to evaluate the interaction of health/infection condition and meloxicam administration for the pharmacokinetic parameters. The interaction of health/infection condition, meloxicam and concentrations of danofloxacin for the protein binding of danofloxacin was evaluated by a three-way ANOVA. The intergroup differences of the pharmacokinetic parameters and protein binding were analyzed using the post hoc Tukey’s test following the ANOVA (SPSS 22.0, SPSS for Windows, SPSS Inc., Chicago, IL, USA). A *p-*value of <0.05 was considered statistically significant.

### 2.4. Microbiological Analysis

#### 2.4.1. Collection of Bronchoalveolar Lavage Fluid Samples

BAL fluid samples were taken from 98 lambs whose clinical scoring results were 1, 2 or 3 in order to isolate and identify the bacterial agents caused infection in lambs with respiratory infections. The trans-nasal method was used for the collection of BAL fluid. Before the collection of BAL fluid, the nostrils of the lambs were cleaned with an alcohol swab. Following extension of the head and neck of the lamb, a sterile nasogastric probe (3.33 × 1210 mm; Bıcakcilar, Istanbul, Turkey) was advanced trans-nasally until a weak resistance was encountered in the trachea. With the recurrent cough reflex, it was understood that the hull area was reached. Then, the catheter was withdrawn 1–2 cm, and 10 mL of sterile saline (0.9% NaCl) was injected into the trachea with the help of an injector placed at the tip of the catheter, followed by immediate aspiration. The BAL fluid (1–2 mL) was kept at 4 °C until microbiological analysis. The microbiological isolation process was started within 12 h following the sampling.

#### 2.4.2. Bacterial Isolation and Identification

Direct streaking was performed on 5% sheep blood agar and MacConkey agar (Merck, Darmstadt, Germany) plates from the BAL fluid samples. The plates were incubated aerobically at 37 °C for 24–48 h. For the *Mycoplasma* culture, the samples were inoculated on *Mycoplasma*-selective agar (*Mycoplasma* agar base, Oxoid, CM0401 + *Mycoplasma*-selective supplement G, Oxoid, SR0059C). The *Mycoplasma* agar plates were incubated at 37 °C for 2–7 days in an environment with 5% CO_2_. The *Mycoplasma*-selective agar plates were examined daily under a microscope after 48 h incubation and were evaluated for growth. Isolated bacteria were identified by colony morphology, growth characteristics, and biochemical tests [47]. The isolated strains were stored at −80 °C for antimicrobial susceptibility tests.

#### 2.4.3. Minimum Inhibitory Concentration

In this study, the broth microdilution method described by the Clinical and Laboratory Standards Institute [48] was used to determine the MIC values of danofloxacin on the bacteria isolated from lambs with respiratory infections. *E. coli* ATCC 25922 and *Staphylococcus aureus* ATCC 29213 were used as quality control isolates. Danofloxacin was tested in a range from 0.016 to 16 µg/mL in doubling dilutions. The final test concentration of bacteria was 5 × 10^5^ colony forming units/mL in Mueller–Hinton broth (Merck, Germany). The MICs were read after 24 h incubation at 37 °C. The lowest concentration of drug that visibly inhibited bacterial growth was accepted as the MIC. The concentrations that inhibited 50% (MIC_50_) and 90% (MIC_90_) of the isolates were calculated for danofloxacin. The MIC_50_ and MIC_90_ were calculated using “*n* × 0.5” and “*n* × 0.9”, respectively, in which *n* was the number of test strains. Then, if the resulting number was not an integer, the next integer following the respective value was taken as the MIC_50_ or MIC_90_ value [49].

#### 2.4.4. Pharmacokinetic/Pharmacodynamic Integration

The PK/PD surrogate indices used to determine the inhibitory activity or effect of danofloxacin against susceptible pathogens were AUC_0–24_/MIC and C_max_/MIC ratios [50,51,52,53]. In this study, the *ƒ*AUC_0–24_/MIC and *ƒ*C_max_/MIC ratios were calculated using the *ƒ*AUC and *ƒ*C_max_ obtained from the free concentrations of danofloxacin in the plasma of the ID and IDM groups after danofloxacin administration and the MIC values determined for the bacterial strains isolated from the lambs with respiratory infections.

## 3. Results

### 3.1. Pharmacokinetics

**Figure 1:**
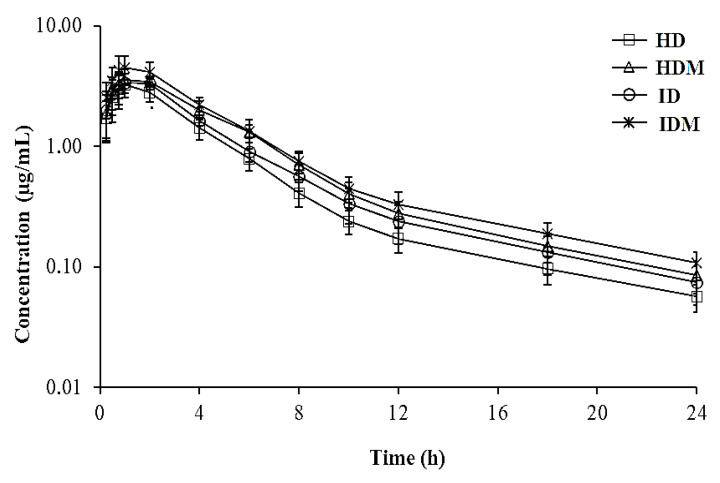
Semi-logarithmic plasma concentration–time curves after subcutaneous administration of danofloxacin alone or co-administered with meloxicam in healthy lambs (*n* = 6) and lambs with respiratory infections (*n* = 7). HD, healthy group who received danofloxacin alone; HDM, healthy group who received danofloxacin and meloxicam; ID, infected group who received danofloxacin alone; IDM, infected group who received danofloxacin and meloxicam. Data are presented as mean ± SD.

Semi-logarithmic plasma concentration–time curves of danoflaxacin alone or co-administered with meloxicam in healthy lambs and lambs with respiratory infections are presented in Figure 1. In the HD, HDM, ID and IDM groups, the plasma concentration of danofloxacin following administration of a 6 mg/kg dose of SC was 1.72 ± 0.64, 1.90 ± 0.77, 2.01 ± 0.84, and 2.56 ± 0.85 µg/mL, respectively, at the first observational point (15 min). Then, it dropped to 0.057 ± 0.015, 0.086 ± 0.026, 0.075 ± 0.027, and 0.108 ± 0.026 µg/mL, respectively, at 24 h.

The pharmacokinetic parameters of danofloxacin alone or co-administered with meloxicam in healthy lambs and lambs with respiratory infections are presented in Table 2. A two-way ANOVA revealed no significant interaction between the healthy/infected conditions and meloxicam administration of any of the pharmacokinetic parameters (*p* > 0.05), but significant main effects were found for the healthy/infected conditions (*p* < 0.05) on Cl_T_/F and AUC and for meloxicam administration (*p* < 0.05) on the Cl_T_/F, Vd_area_/F, Vd_ss_/F, C_max_, and AUC, indicating that these factors acted independently. Danofloxacin exhibited similar t_1/2ʎz_ and MRT in all groups (*p* > 0.05). However, the Cl_T_/F and Vd_area_/F of danofloxacin showed significant differences between the groups (*p* < 0.05). The Cl_T_/F decreased significantly (*p* < 0.05) upon meloxicam administration (HDM and IDM groups) and the effect of infection (ID and IDM groups). The administration of meloxicam to healthy (HDM) and infected lambs (IDM) significantly increased the Vd_area_/F and Vd_ss_/F of danofloxacin compared to the HD group (*p* < 0.05). In the HD, HDM, ID, and IDM groups, danofloxacin reached different C_max_ values of 3.36, 3.73, 3.61, and 4.59 µg/mL (*p* < 0.05), respectively, with T_max_ values of 1.0 h (*p* > 0.05). In particular, the administration of meloxicam (IDM) to infected lambs significantly increased the C_max_ of danofloxacin compared to the HD group (*p* < 0.05). The AUC increased significantly in the HDM and IDM groups compared to the HD and ID groups (*p* < 0.05). In the ID and IDM groups, the *ƒ*AUC was 10.36 (7.43–14.99) h·µg/mL and 14.80 (11.25–20.33) h·µg/mL, respectively, and *ƒ*C_max_ was 2.42 (2.00–3.00) µg/mL and 3.60 (2.58–5.05) µg/mL, respectively.

### 3.2. In Vitro Protein Binding of Danofloxacin

The protein binding ratio of danofloxacin alone or in combination with meloxicam in plasma samples of healthy lambs and lambs with respiratory infections is presented in Table 3. The protein binding ratio (mean ± SD) of danofloxacin for PBHD, PBHDM, PBID and PBIDM groups were 32.0 ± 4.8%, 22.7 ± 3.1%, 32.8 ± 3.9% and 21.6 ± 4.0%, respectively. No interaction of the health/infection condition, meloxicam and concentration of danofloxacin for the protein binding of danofloxacin was determined by the three-way ANOVA, but the meloxicam and concentration of danofloxacin acted independently. No significant effects on the plasma protein binding of danofloxacin were found for the healthy/infected conditions (*p* > 0.05). When danofloxacin was in combination with meloxicam (PBHDM and PBIDM groups), the plasma protein binding of danofloxacin decreased significantly (*p* < 0.05). In PBHD group, the protein binding ratio at the concentration of 5 µg/mL is statistically lower than that at the concentration of 0.05 µg/mL (*p* < 0.05). Preliminary studies found negligible binding (<1%) of danofloxacin to the ultrafiltration device.

### 3.3. Pharmacodynamics

In the BAL fluid samples taken from the infected lambs, the *E. coli* (*n* = 6), *Streptococcus* spp. (*n* = 3), and *M. haemolytica* (*n* = 83) strains were isolated and identified. No growth was detected on the *Mycoplasma* cultures. The MIC values of danofloxacin were determined as 0.063–0.125 μg/mL for the *E. coli* strain and >16 μg/mL for the *Streptococcus* spp. strain. The breakpoints of danofloxacin for *E. coli* and *Streptococcus* spp. have not been defined. However, all isolates of *E. coli* isolated from the lambs could be susceptible to danofloxacin according to the susceptibility breakpoint (8 µg/mL) of danofloxacin reported for *E. coli* isolated from pigs [54]. The MIC_90_ value could not be determined for the *E. coli* or *Streptococcus* spp. strains due to the insufficient number of isolates.

**Figure 2:**
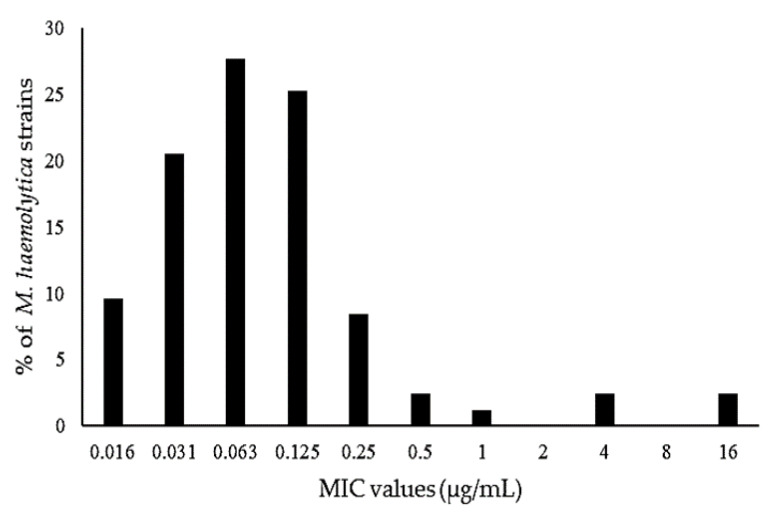
Minimum inhibitory concentration (MIC) distribution of danofloxacin in the 83 strains of *M. haemolytica.* The MIC_50_ and MIC_90_ were set at 0.063 and 0.25 μg/mL, respectively.

In this study, the MIC values of danofloxacin for the 83 *M. haemolytica* isolates isolated from the lambs ranged from 0.016 to >16 µg/mL. The percent MIC distribution of danofloxacin for the 83 isolates of *M. haemolytica* is presented in Figure 2. The percent MIC distribution for the concentrations of 0.016, 0.031, 0.063, 0.125, 0.25, 0.5, 1, 4, and 16 μg/mL was 9.6%, 20.5%, 27.7%, 25.3%, 8.4%, 2.4%, 1.2%, 2.4%, and 2.4%, respectively. There are no CLSI-approved breakpoint MIC values of danofloxacin for *M. haemolytica* isolated from sheep. The susceptible (0.25 µg/mL), intermediate (0.5 µg/mL), and resistant (1 µg/mL) MIC breakpoint values for *M. haemolytica* isolated from cattle have been determined [55,56]. For *M. haemolytica* isolated from the lambs, the MIC_50_ value was 0.063 µg/mL and the MIC_90_ value was 0.25 µg/mL, in which 91.6% of the population was below the susceptible breakpoint of 0.25 µg/mL.

### 3.4. Pharmacokinetic/Pharmacodynamic Integration

The PK/PD surrogate indices determined using the pharmacokinetic parameters obtained from the free concentrations of danofloxacin and the MIC values determined for the bacterial strains isolated from the lambs with respiratory infections are presented in Table 4. In the ID and IDM groups, the AUC_0–24_/MIC_90_ estimated using the in vitro MIC_90_ data (0.25 µg/mL) for *M. haemolytica* and the in vivo pharmacokinetic parameters of danofloxacin was 41.44 h and 59.21 h, while the *ƒ*C_max_/MIC_90_ was 9.69 and 14.40, respectively. In the ID and IDM groups, the *ƒ*AUC_0–24_/MIC for the *E. coli* isolates ≤0.125 µg/mL was 82.89 h and 118.43 h, while the *ƒ*C_max_/MIC was 19.39 and 28.80, respectively. The PK/PD surrogate markers for the *Streptococcus* spp. isolates with an MIC value >16 µg/mL were not determined.

## 4. Discussion

### 4.1. Pharmacokinetics of Danofloxacin

Danofloxacin has been recommended for use at a dose of 6 mg/kg against *M. haemolytica* isolates with an MIC value of 0.25 μg/mL by PK/PD modeling in sheep [51,57]. In addition, the clinical efficacy of a 6 mg/kg dose of danofloxacin has been demonstrated in an experimental infection caused by *M. haemolytica* in lambs [17]. In this study, the injection route (SC) and dose (6 mg/kg) of danofloxacin administered to lambs were determined based on previous studies [17,50,56]. Meloxicam was administered at the approved dose (1 mg/kg) in sheep [20].

In this study, danofloxacin exhibited a similar t_1/2ʎz_ in all groups, including the healthy and infected lambs. The t_1/2ʎz_ is a hybrid parameter affected by changes in the Vd and Cl of the drug [58]. In this study, the V_darea_/F and Cl_T_/F values of danofloxacin significantly decreased with the administration (HDM and IDM groups) of meloxicam and the effect (ID and IDM groups) of infection. However, the decrease in both the Vd_area_/F and Cl_T_/F values caused by the infection and the administration of meloxicam may be the reason for similar t_1/2ʎz_ parameters in all groups. These results are also consistent with the results of studies in which the Cl_T_ and Vd of enrofloxacin decreased, but its t_1/2_ did not change [30,59].

In this study, the Cl_T_/F of danofloxacin significantly decreased with the administration (HDM and IDM groups) of meloxicam and the effect (ID and IDM groups) of infection. The lower Cl_T_/F of danofloxacin in the HDM (0.32 L/h·kg) and IDM (0.28 L/h·kg) groups compared to the HD (0.45 L/h·kg) and ID (0.38 L/h·kg) groups indicates that danofloxacin interacts with meloxicam. The elimination of danofloxacin and meloxicam has not been observed in sheep. The excretion of danofloxacin occurs approximately equally in urine and feces; while approximately 80% of the drug is removed unchanged, 20% of the drug is removed as a β-glucuronide conjugate of danofloxacin, desmethyldanofloxacin and danofloxacin-*N*-oxide, which are formed as a result of phase I and extensive phase II reactions [60]. Meloxicam is metabolized extensively in the liver through phase I reactions (conjugate derivative has not been determined) and less than <10% as unchanged meloxicam is excreted in the urine [61,62]. These data may indicate that danofloxacin and meloxicam interact primarily at the kidney level due to differences in their metabolism pathways. It is known that NSAIDs reduce the renal blood flow and glomerular filtration rate by inhibiting the synthesis of vasodilator prostaglandin (PG)E_2_ and PGI_2_, which play a role in maintaining normal kidney function [63,64]. PGs are synthesized in the kidneys by both COX-1 and COX-2 enzymes. Selective COX-2 inhibitors have been shown to have similar effects on renal hemodynamics as non-selective NSAIDs [65,66]. The effect of meloxicam, a selective COX-2 inhibitor, on the Cl_T_/F of danofloxacin may be related to its decrease in renal blood flow and glomerular filtration rate.

Meloxicam is a substrate of BCRP, a member of the ATP-dependent efflux transporters [67,68]. Danofloxacin is the substrate of P-gp and MRP2, in addition to BCRP [16,43]. In sheep, ivermectin reduces the passage of danofloxacin entering the milk by a BCRP-mediated interaction (40%) [43]. Ciprofloxacin, a substrate of BCRP but not of P-gp or MRP2, reduces the secretion and absorption of danofloxacin in a Caco-2 cell model [16]. In this study, the decrease in the Cl_T_/F of danofloxacin in the IDM and HDM groups may have resulted from the limitation of the excretion of danofloxacin due to a BCRP-mediated danofloxacin–meloxicam interaction in the excretion routes, such as the intestines, bile duct, or proximal tubule cells.

In this study, the lower Cl_T_/F of danofloxacin in the ID (0.38 L/h·kg) and IDM (0.28 L/h·kg) groups than that in the HD (0.45 L/h·kg) and HDM (0.32 L/h·kg) groups indicates the effect of infection. These findings are consistent with other studies that reported a reduced Cl_T_/F of danofloxacin, marbofloxacin, and tilmicosin in respiratory infections [69,70,71,72]. However, the Cl_T_/F of marbofloxacin increased in lambs naturally infected with *M. haemolytica* [73]. The Cl_T_ of drugs is influenced by a variety of factors, particularly the blood flow to the excretion organs, the binding ratio of the drug to plasma proteins, and the activity of drug-metabolizing enzymes. These factors can be affected by sepsis and septic shock and may alter drug excretion and metabolism, leading to a reduced Cl_T_ [74,75]. It is reported that inflammation and infection could lead to downregulation of drug-metabolizing enzymes and transporters (primarily of the ABC superfamily), resulting in higher plasma concentrations and altering some distribution processes. Many inflammatory mediators (interleukines, cytokines, transforming growth factor, tumor necrosis factor or interferons) reduced the gene expression of cytochrome P450 complex in liver. On the other hand, inflammation and infection also downregulate drug efflux transporters ATP-binding cassette (ABC) superfamily. These transporters are ubiquitous in the organism and play a major role in drug transport of antimicrobials, among other drugs. A specific ABC transporter, the BCRP, and ABCG2 family transporter, is present in the human mammary gland and is responsible to excrete fluoroquinolones to milk. BCRP was present in mammary glands of animals including sheep and goats, and it is reported that danofloxacin is a substrate of BCRP, and inflammation and infection could lead to a downregulation of this transporter, affecting the disposition of the drug in the organism [76,77,78,79,80]. In lambs infected with *M. haemolytica*, the release of pro-inflammatory cytokines such as interleukin-1β, interleukin-8, and tumor necrosis factor-alpha has been shown to increase [81]. Pro-inflammatory cytokines reduce the activities of various cytochrome P450 and phase II enzymes, as well as the expression and function of transporter proteins such as P-gp, BCRP, and MRP2, which play an important role in the pharmacokinetics of drugs [82,83,84,85]. These effects have been reported as the cause for the decreased Cl_T_ of danofloxacin in infected animals [16,86]. In this study, the decreased Cl_T_/F of danofloxacin in the ID group may be related to changes in blood flow into the excretion organs caused by infection and the decrease in the functions of phase II enzymes and the P-gp, BCRP, and MRP2 transporters. The partial decrease in the Cl_T_/F of danofloxacin in the IDM group compared to the HDM group may be due to the therapeutic effect of meloxicam on the inflammation and fever caused by infection in the infected lambs.

In this study, the Vd_area_/F and Vd_ss_/F of danofloxacin were significantly decreased in the HDM and IDM groups compared to the HD group. These data show that the Vd_area_/F and Vd_ss_/F decreased with the administration of meloxicam (HDM and IDM). Similarly, the Vd of enrofloxacin and moxifloxacin has been shown to decrease with upon administration of some NSAIDs in healthy animals [30,31]. The protein binding ratio of danofloxacin in HD, HDM, ID and IDM groups were 32.0 ± 4.8%, 22.7 ± 3.1%, 32.8 ± 3.9% and 21.6 ± 4.0%, respectively. The binding ratio of meloxicam to plasma proteins is high (96% in dogs and 98% in calves) [60]. In this study, meloxicam decreased significantly the plasma protein binding of danofloxacin in HDM and IDM groups. A decrease in the binding ratio of danofloxacin to plasma proteins can be expected to cause an increased Vd. However, an increased concentration of danofloxacin in the plasma due to its reduced elimination by meloxicam can lead to a low calculation of Vd/F. Another reason for the decreased Vd/F in the HDM and IDM groups may be the limitation of the body disposition of danofloxacin, which is a substrate of P-gp, MRP2, and BCRP, by meloxicam [16,43,87]. Danofloxacin reaches 4 times higher concentrations in the lungs, bronchial mucosa, and bronchial secretions and 10 times higher in the milk than that in the plasma [12,57,88]. ABC transporters such as P-gp, MRP2, and especially BCRP are responsible for the high concentrations of danofloxacin in the lung tissues and milk [16,43]. The limitation of the excretion and passage of danofloxacin to highly accumulated tissues such as the lungs due to a BCRP-mediated danofloxacin–meloxicam interaction may be the reason for the decreased Vd/F in the HDM and IDM groups. In particular, given that the interest lies with the influence of meloxicam in the pharmacokinetics of danofloxacin, a more robust approach would be to also analyze the concentration–time profile of meloxicam to examine whether there is a potential concentration-dependent effect.

The rate of drug absorption from the site of administration is affected by conditions such as septicemia and septic shock, which affect the regional blood flow [74,89]. A systemic inflammatory response has been reported in pneumonia caused by *M. haemolytica* [81]. In this study, an increase (>40.5 °C) in body temperature, indicating the systemic effects of the infection, was determined in infected lambs. Therefore, the absorption rate of danofloxacin can be expected to change following SC administration in infected lambs. However, in this study, the C_max_/AUC and T_max_ parameters used to evaluate the rate of drug absorption from the site of administration [90] were similar between the groups.

In this study, the C_max_ of danofloxacin in the HD, HDM, ID, and IDM groups was 3.42, 3.76, 3.64, and 4.72 µg/mL, respectively. The administration of meloxicam (IDM) to infected lambs significantly increased the C_max_ of danofloxacin compared to the HD group. The AUC of danofloxacin increased significantly in the HDM and IDM groups compared to in the HD and ID groups. The absorption extent, Cl, and Vd of a drug contribute to the formation of C_max_ and AUC [91,92]. Danofloxacin, following SC administration, in sheep has been shown to achieve a high bioavailability of 94% [51,93]. In this study, the decreased Vd_area_/F and Cl_T_/F in the HDM and IDM groups may lead to the formation of a high C_max_ and AUC. A high C_max_ and AUC has been reported for enrofloxacin co-administered with flunixin meglumine and for danofloxacin in calves with respiratory tract infections [35,70]. Danofloxacin has been shown to demonstrate linear pharmacokinetics at doses of 1.25–10 mg/kg following SC administration and no toxic symptoms at doses of 10 and 20 mg/kg for six days or doses of 18 and 24 mg/kg for three days in calves [94]. In this study, a 6 mg/kg dose of danofloxacin in the IDM group created a high C_max_ within the therapeutic range. This may have resulted in an increased therapeutic effect of danofloxacin in the IDM group.

### 4.2. Pharmacokinetic/Pharmacodynamic Integration

The PK/PD surrogate parameters showing the efficacy of fluoroquinolones are the *ƒ*AUC_0–24_/MIC and *ƒ*C_max_/MIC ratios [95,96]. In the treatment of infections caused by Gram-negative bacteria, an *ƒ*AUC_0–24_/MIC ratio should be 100–125 and a *ƒ*C_max_/MIC ratio should be 8–10 in order to achieve an ideal bactericidal effect and reduce the risk of resistance [97,98]. In in vitro studies, it has been shown that while fluoroquinolone dosages with a *ƒ*C_max_/MIC ratio of >3 cause a 99% reduction in the number of bacteria, dosages with a *ƒ*C_max_/MIC ratio of ≥8 are sufficient to prevent the emergence of resistant bacteria [99]. In the present study, the *ƒ*AUC_0–24_/MIC and *ƒ*C_max_/MIC ratios were calculated only for the ID and IDM groups. In the ID and IDM groups, the *ƒ*AUC_0–24_/MIC ratio of danofloxacin for susceptible *E. coli* isolates with an MIC value of 0.125 µg/mL was 82.89 h and 118.43 h, respectively, while the *ƒ*C_max_/MIC ratio was 19.39 and 28.80, respectively. Danofloxacin in the ID and IDM groups provided the ideal *ƒ*C_max_/MIC ratios following SC administration at a dose of 6 mg/kg. However, the ideal *ƒ*AUC_0–24_/MIC ratio was obtained for the IDM group only.

A susceptible MIC breakpoint of ≤0.25 µg/mL for danofloxacin against *M. haemolytica* has been reported [56,100]. In this study, the MIC_90_ value of danofloxacin for *M. haemolytica* isolates isolated from lambs with respiratory infections was 0.25 µg/mL. In the ID and IDM groups, the *ƒ*AUC_0–24_/MIC_90_ ratio was 41.44 h and 59.21 h, respectively, while the *ƒ*C_max_/MIC_90_ ratio was 9.69 and 14.40. Danofloxacin at a dose of 6 mg/kg achieved the ideal *ƒ*C_max_/MIC_90_ value of 8–10 but did not reach the *ƒ*AUC_0–24_/MIC_90_ ratio of ≥125 h previously reported for fluoroquinolones [95,96]. However, studies have reported that the optimal *ƒ*AUC_0–24_/MIC_90_ value is not an absolute value, and the AUC_0–24_/MIC_90_ value of danofloxacin, which can provide bacterial elimination of *M haemolytica*, is 2–4 times lower than 125 h [50,51,52,101]. The ideal ex vivo and in vivo AUC_0–24_/MIC values of danofloxacin for the elimination of *M. haemolytica* isolates from sheep have been reported to be 28.7 and 55.9 h, respectively [51]. When evaluated on the basis of these data, danofloxacin provided the ideal in vivo *ƒ*AUC_0–24_/MIC_90_ ratio (55.9 h) for *M. haemolytica* isolates with MIC values of ≤0.25 µg/mL in the ID and IDM groups, but not in the ID group. Furthermore, previous studies have shown that danofloxacin in the lung tissues and bronchial mucosa reaches approximately 5- and 3-times higher concentrations, respectively, than those obtained in the plasma [11]. Therefore, in the present study, the concentration of danofloxacin obtained in the lung tissues and bronchial mucosa of the lambs in the ID group may have significantly exceeded the MIC values required for the elimination of *M. haemolytica*, which is an extracellular localization [70,102].

## 5. Conclusions

The administration of meloxicam and the presence of infection in lambs reduced the Vd/F and Cl_T_/F of danofloxacin but did not affect the t_1/2λz_. Meloxicam increased the plasma concentration of danofloxacin in both healthy lambs and lambs with respiratory infections. In lambs with respiratory infections, an increased plasma concentration of danofloxacin within the therapeutic range may result in a better therapeutic effect. However, the clear definition of an increase in the therapeutic effect requires the determination of the concentration of danofloxacin in the target tissues and the effects of the resulting concentration on bacterial eradication. In addition, repeated doses of danofloxacin are required for the treatment of infection. A danofloxacin–meloxicam interaction may not be predicted following administration at repeated doses. Therefore, it is necessary to determine the pharmacokinetics of danofloxacin and meloxicam following the administration of repeated doses before simultaneous use in lambs with respiratory infections.

When co-administration with meloxicam, danofloxacin following SC administration at a dose of 6 mg/kg in lambs with respiratory infections provided optimum AUC_0–24_/MIC (>56 h) and C_max_/MIC (>8) values for susceptible *M. haemolytica* isolates with an MIC_90_ value of 0.25 µg/mL and for susceptible *E. coli* isolates with an MIC value of ≤0.125 µg/mL. However, due to bacterial isolation in a small number of samples in this study, the determination of pharmacodynamic data on bacterial agents isolated from more lambs with respiratory infections will be more valuable for the rational use of danofloxacin.

## Figures and Tables

**Table 1:** The mean (±SD) age, body weight, rectal temperature, and respiratory rate of the healthy lambs (*n* = 6) and those with respiratory infections (*n* = 7) selected for the pharmacokinetic study. HD, healthy group who received danofloxacin alone; HDM, healthy group who received danofloxacin and meloxicam; ID, infected group who received danofloxacin alone; IDM, infected group who received danofloxacin and meloxicam.

Parameters	HD	HDM	ID	IDM
Age (month)	3.20 ± 0.23	3.25 ± 0.36	3.50 ± 0.30	3.54 ± 0.51
Body weight (kg)	27.50 ± 3.03	28.17 ± 3.27	28.86 ± 3.08	27.86 ± 3.44
Rectal temperature (°C)	39.77 ± 0.15	39.93 ± 0.12	41.01 ± 0.22	41.04 ± 0.22
Respiratory rate (breath/min)	42.17 ± 3.63	42.83 ± 2.79	61.00 ± 3.27	62.43 ± 3.46

**Table 2:** The pharmacokinetic parameters of danofloxacin (6 mg/kg; SC) alone or co-administered with meloxicam (1 mg/kg; SC) in healthy lambs (*n* = 6) and lambs with respiratory infections (*n* = 7). t_1/2ʎz_, terminal half-life; Cl_T_/F, total clearance; Vd_area_/F, volume of distribution; Vd_ss_/F, volume of distribution in a steady state; T_max_, time to reach the peak concentration; C_max_, peak plasma concentration; AUC, area under the concentration–time curve; MRT, mean residence time; HD, healthy group who received danofloxacin alone; HDM, healthy group who received danofloxacin and meloxicam; ID, infected group who received danofloxacin alone; IDM, infected group who received danofloxacin and meloxicam.

Parameters	HD	HDM	ID	IDM
t_1/2ʎz_ (h)	6.98 (5.75–8.15)	6.46 (4.82–8.24)	6.07 (5.01–7.48)	6.73 (5.14–8.12)
Cl_T_/F (L/h·kg)	0.45 (0.39–0.51) ^a^	0.32 (0.28–0.36) ^b,c^	0.38 (0.31–0.42) ^b^	0.28 (0.21–0.31) ^c^
Vd_area_/F (L/kg)	4.55 (3.52–5.40) ^a^	3.02 (2.01–4.18) ^b^	3.35 (2.98–4.20) ^a,b^	2.73 (1.94–3.44) ^b^
Vd_ss_/F (L/kg)	2.46 (1.94–3.23) ^a^	1.88 (1.39–2.24) ^b^	2.12 (1.61–2.51) ^a,b^	1.70 (1.23–2.21) ^b^
T_max_ (h)	1.01 (0.75–2.00)	1.25 (1.00–2.00)	1.34 (1.00–2.00)	1.23 (0.75–2.00)
C_max_ (µg/mL)	3.36 (2.46–4.46) ^b^	3.73 (2.89–4.30) ^a,b^	3.61 (2.97–4.46) ^a,b^	4.59 (3.29–6.44) ^a^
AUC_0–24_ (h.µg/mL)	12.68 (11.04–14.61) ^c^	17.69 (15.94–20.32) ^a,b^	14.99 (13.59–17.97) ^b,c^	20.23 (18.37–25.93) ^a^
AUC_0–∞_ (h·µg/mL)	13.26 (11.63–15.26) ^c^	18.52 (16.56–20.73) ^a,b^	15.65 (13.96–19.06) ^b,c^	21.30 (19.19–27.29) ^a^
C_max_/AUC	0.25 (0.20–30)	0.20 (0.15–0.25)	0.23 (0.17–0.29)	0.22 (0.17–27)
MRT (h)	5.43 (4.54–6.27)	5.81 (4.79–6.95)	5.54 (3.86–6.78)	6.02 (4.72–7.51)

Data are presented as the geometric mean (min–max) and were analyzed by two-way ANOVA with Tukey’s test. ^a,b,c^ Different letters in the same row are statistically different (*p* < 0.05).

**Table 3:** Protein binding ratio (%) of danofloxacin alone or in combination with meloxicam in plasma samples of healthy lambs and lambs with respiratory infections. PBHD, healthy group who was spiked with danofloxacin alone; PBHDM, healthy group who was spiked with danofloxacin and meloxicam; PBID, infected group who was spiked danofloxacin alone; PBIDM, infected group who was spiked danofloxacin and meloxicam.

Danofloxacin Concentration (µg/mL)	PBHD	PBHDM	PBID	PBIDM
0.05	36.3 ± 2.1 ^a^	24.3 ± 2.1 ^b^	36.0 ± 3.6 ^a^	22.0 ± 3.0 ^b^
0.5	32.0 ± 4.6 ^a^	21.0 ± 3.0 ^b^	33.0 ± 1.0 ^a^	24.3 ± 3.8 ^a,b^
5.0	27.7 ± 3.1 ^a,^*	22.6 ± 4.0 ^a,b^	29.3 ± 3.5 ^a^	18.3 ± 3.5 ^b^
Mean ± SD	32.0 ± 4.8 ^a^	22.7 ± 3.1 ^b^	32.8 ± 3.9 ^a^	21.6 ± 4.0 ^b^

Data are presented as the mean ± SD and were analyzed by three-way ANOVA with Tukey’s test. * In PBHD group, the protein binding ratio at the concentration of 5 µg/mL is statistically lower than that at the concentration of 0.05 µg/mL (*p* < 0.05). ^a,b^ Different letters in the same row are statistically different (*p* < 0.05).

**Table 4:** The PK/PD surrogate indices determined using the pharmacokinetic parameters obtained from the free concentrations of danofloxacin and the MIC values determined for the bacterial strains isolated from the lambs with respiratory infections. MIC, minimum inhibitor concentration; AUC, area under the concentration–time curve; C_max_, peak plasma concentration; GM, geometric mean; ID, infected group who received danofloxacin alone; IDM, infected group who received danofloxacin and meloxicam.

Animal No	*ƒ*AUC_0–24_/MIC		*ƒ*C_max_/MIC	
	ID	IDM	ID	IDM
*Mannheimia haemolytica,* MIC_90_ of 0.25 µg/mL				
1	38.42	57.62	7.99	10.32
2	37.83	44.98	10.89	13.35
3	36.50	81.31	9.34	20.21
4	29.70	59.95	11.99	11.26
5	50.42	56.37	10.43	19.17
6	59.97	58.85	8.66	14.76
7	44.08	60.90	9.15	14.45
GM (min–max)	41.44 (29.70–59.97)	59.21 (44.98–81.31)	9.69 (7.99–11.99)	14.40 (10.32–20.21)
***Escherichia coli*** **, MIC of 0.125 µg/mL**				
1	76.84	115.23	15.98	20.65
2	75.66	89.96	21.78	26.69
3	73.01	162.63	18.69	40.42
4	59.40	119.89	23.98	22.53
5	100.83	112.74	20.86	38.33
6	119.94	117.71	17.31	29.53
7	88.16	121.80	18.30	28.90
GM (min–max)	82.89 (59.40–119.94)	118.43 (89.96–162.63)	19.39 (15.98–23.98)	28.80 (20.65–40.42)

## Data Availability

The data presented in this study are available on request from the corresponding author.
